# Canopy Homolog 2 as a Novel Molecular Target in Hepatocarcinogenesis

**DOI:** 10.3390/cancers13143613

**Published:** 2021-07-19

**Authors:** Anna Kakehashi, Shugo Suzuki, Masayuki Shiota, Nina Raymo, Min Gi, Taro Tachibana, Vasily Stefanov, Hideki Wanibuchi

**Affiliations:** 1 Department of Molecular Pathology, Osaka City University Graduate School of Medicine, Abeno-ku, 1-4-3 Asahi-machi, Osaka 545-8585, Japan; suzuki.shugo@med.osaka-cu.ac.jp (S.S.); nraymo21@colby.edu (N.R.); mwei@med.osaka-cu.ac.jp (M.G.); wani@med.osaka-cu.ac.jp (H.W.); 2 Department of Molecular Biology of Medicine, Osaka City University Graduate School of Medicine, 1-4-3 Asahi-machi, Abeno-ku, Osaka 545-8585, Japan; sio@med.osaka-cu.ac.jp; 3 Department of Bioengineering, Graduate School of Engineering, Osaka City University, 3-3-138 Sugimoto, Sumiyoshi-ku, Osaka 558-8585, Japan; taro-tachibana@osaka-cu.ac.jp; 4 Department of Biochemistry, Faculty of Biology, Saint Petersburg State University, 199034 Saint Petersburg, Russia; v.stefanov@spbu.ru

**Keywords:** CNPY2, hepatocarcinogenesis, mice, human, molecular target, prognostic marker

## Abstract

**Simple Summary:**

Protein canopy homolog 2 (CNPY2) controls the outgrowth of neurites through positive regulation of their outgrowth in neuroblastoma and pheochromocytoma due to stabilizing the myosin regulatory light chain (MRLC). Given the important role in endoplasmic reticulum (ER) stress and proteolysis, we focused on investigation of CNPY2 expression in mouse and human hepatocarcinogenesis and its clinical relevance in virus-associated liver cancer. CNPY2 elevation and coordinated accumulation of numerous cytoskeletal proteins and those involved in ER and mitochondrial stresses was found in mouse altered foci and tumors by proteome analysis of microdissected lesions. In Huh7 and HepG2 human liver cancer cells, CNPY2 increase was significantly associated with cell cycle progression, activated cell proliferation and invasion. CNPY2 expression was high in HCV-associated HCC patients, being positively associated with survival. To the best of our knowledge, this is the first report to demonstrate that CNPY2 may become a useful prognostic biomarker in HCV-associated HCC.

**Abstract:**

In the present study, the role of a novel protein involved in neurite development and endoplasmic reticulum (ER) stress, canopy homolog 2 (CNPY2), was investigated in mouse and human hepatocarcinogenesis. Firstly, a sensitive quantitative and qualitative detection of protein expression using QSTAR Elite LC-Ms/Ms was performed for the analysis of lysates of microdissected hepatocellular altered foci (AF), adenomas (HCAs), carcinomas (HCCs) and peri-tumoral livers from C57Bl/6J mice treated with diethylnitrosamine (DEN) and then maintained for 27 or 38 weeks on basal diet. Significant overexpression of 18.5 kDa CNPY2 processed form was demonstrated in AF, HCAs and HCCs, while low expression was observed in the livers of DEN-treated and control mice. Furthermore, CNPY2 elevation in AF and tumors was coordinated with accumulation of numerous cytoskeletal proteins, including cytokeratins 8 and 18, actin, non-muscle myosin and septin 9 and those involved in ER and mitochondrial stresses such as calreticulin, prohibitins 1 and 2 and YME1-like-1. Knockdown of CNPY2 in Huh7 and HepG2 human liver cancer cells resulted in significant suppression of cell survival and invasive potential, inhibition of cyclin D1, induction of p21^Waf1/Cip1^ and suppression of the apoptosis inhibitor Bcl2. In contrast, transfection of a mouse CNPY2 (mCNPY2-Ds-Red) vector plasmid in Huh7 and HepG2 cancer cells, with subsequent accumulation of CNPY2 in the ER, resulted in significant increase in cancer cells survival. Clinicopathological analysis in 90 HCV-positive HCC patients, revealed significant association of CNPY2 overexpression with poor overall (*p* = 0.041) survival. Furthermore, CNPY2 increase was associated with vessel invasion (*p* = 0.038), poor histological differentiation (*p* = 0.035) and advanced clinical stage (*p* = 0.016). In conclusion, CNPY2 is a promising molecular target elevated early in hepatocarcinogenesis and prognostic marker for human HCV-associated HCC. CNPY2 is involved in the processes of ER stress, cell cycle progression, proliferation, survival and invasion of liver tumor cells.

## 1. Introduction

Hepatocellular carcinoma (HCC), the fifth most common cancer and the third leading cause of death from cancer worldwide [1], commonly emerges on a background of chronic liver disease with continuous rounds of necrosis and regeneration, inflammation and oxidative stress [2,3]. At the molecular level, large numbers of genetic and/or epigenetic events have been found associated with its development, such as inactivation of the tumor suppressor p53, β-catenin mutations, and methylation of cancer-relevant genes (p16, COX2, etc.) [2,3,4]. Although these have apparent mechanistic significance, early carcinogenic events occurring at cellular and molecular levels which are driving the disease pathogenesis still require clarification. 

In our previous studies, we searched for new biomarkers of animal hepatocarcinogenesis and reported the significant overexpression of intermediate filament members, cytokeratins 8 (CK8) and 18 (CK18), and their complex (CK8/18) formation in glutathione-S transferase placental form positive (GST-P^+^) foci and basophilic foci of diethylnitrosamine (DEN)-treated rats and mice, respectively [5,6]. In mice, CK8/18 was chosen as the best candidate biomarker for liver preneoplastic lesions capable of progressing into HCCs, and now is applied in cancer risk assessment for the evaluation of environmental carcinogens, drugs, and food additives safety using mouse models [6]. In rats, CK8/18 overexpression in the cytoplasm of liver preneoplastic cells was accompanied by increase in cell proliferation, elevation of histone type 2 H2aa3, mitochondrial prohibitins 1 (PHB1) and 2 (PHB2) and septin 9 (SEPT9), and serve as an indicator of transformation of GST-P^+^ foci into HCC [7]. However, in humans, the reliable early biomarkers and novel effector molecules which could be applied as therapeutic targets in liver cancer are still highly needed.

Canopy homolog 2 (CNPY2) is a novel protein, which has recently attracted scientists’ attention. It was previously reported to control the outgrowth of neurites, through positive regulation of their outgrowth in neuroblastoma and pheochromocytoma PC12 cells due to stabilizing the myosin regulatory light chain (MRLC) [8], and has been suggested as a novel modulator of cell motility [9]. Furthermore, in neurite, this effect is likely to be related to the prevention of MRLC-interacting protein (MIR)-mediated ubiquitination of myosin and its subsequent proteasomal degradation [8]. In addition, co-localization of human CNPY2 and monkey ezrin-radixin-moesin (ERM)-like MIR proteins have been reported to occur in intracellular spaces, such as with COS-7 cells and in primary neurons [8]. These processes could induce dynamic interactions between the actin cytoskeleton and specific proteins, crucial for changes in cell shape and motility. However, CNPY2 roles have not yet been fully investigated in mouse and human hepatocarcinogenesis. The detailed study is needed to elucidate its involvement in processes of liver tumor promotion and progression. Therefore, we investigated here alterations of CNPY2 expression in mouse liver preneoplastic lesions, tumors, and HCCs of HCV-positive (HCV^+^) patients. In animal experiments, sensitive quantitative and qualitative assessment of protein expression in preneoplastic lesions, tumors and non-tumor mouse liver tissues using the proteome and bioinformatic analyses was applied. Furthermore, in vitro functional and clinicopathological analyses were carried out to investigate CNPY2 role in human liver cancer and its association with HCV^+^ HCC patients survival and clinicopathological variables. 

## 2. Results

### 2.1. Liver Samples and Profiling by QSTAR-Elite LC-Ms/Ms

Formalin-fixed and paraffin embedded (FFPE) serial liver sections with altered foci (AF), hepatocellular adenomas (HCAs) and HCCs were prepared using liver samples from our previous experiment, in which development of liver preneoplastic and neoplastic lesions was observed after DEN administration to 6-week-old C57Bl/6J mice [6]. Significant increase in liver AF number, and incidences and multiplicities of tumors was obvious in the livers of mice 27 and 38 weeks, respectively, after the initiation [6]. In DEN-administered mice, basophilic lesions were found to constitute almost 100% of C57Bl/6J AF. Only few mixed-type foci and no eosinophilic foci were detected. Lesions were microdissected to allow proteome analysis of specific populations. The results of QSTAR Elite LC-Ms/Ms label and non-label proteome analyses of microdissected liver AF and tumors are presented in Table 1. For the identification of proteins participating in the onset and progression of mice hepatocarcinogenesis, we compared the proteomes of microdissected AF (27-week time-point), HCAs and HCCs (38-week time-point), peri-tumoral liver tissue, and normal liver tissue of untreated mice. From microdissected AF, HCAs, and HCCs of mice in the DEN initiation group, 20, 39, and 81 proteins, respectively, were identified with 95% confidence or higher and quantified using ProteinPilot™ 2.0 Software. Marked overexpression of CNPY2 was noted in all types of preneoplastic and neoplastic hepatocellular lesions. Significant increase in the processed form 18.5 kDa-CNPY2 protein in HCCs of DEN-treated mice and low levels in the surrounding and non-treated mice livers was confirmed by Western blot analysis (Figure S1). CNPY2 increase in AF and liver tumors was closely associated with elevation of numerous cytoskeletal proteins such as actins alpha 1 (ACTA1) and gamma 2 (ACTG2), non-muscle myosin, heavy chain 9 (MYH9), tropomyosin 3 (TPM3), septin 9 (SEPT9), plectin (PLEC), cytokeratins 8 (CK8) and 18 (CK18), tubulin, beta 2C (TUBB2C) and fibronectin 1 (FN1), and upregulation of calreticulin (CALR), prohibitins 1 (PHB1) and 2 (PHB2), and YME1-like 1 (YME1L1), participating in regulation of transcription, and ER and mitochondrial stresses. Taking in account observed significant elevation of CNPY2 in mouse AF and liver tumors associated with increase in numerous cytoskeletal and ER-related proteins, low expression in non-treated and surrounding livers, and a known CNPY2 ability to suppress proteolysis promoting ER stress and stabilize actomyosin complexes [10], we proposed that it may play an important role in the processes of tumor growth, cell proliferation, survival, invasion and metastasis. Therefore, in this study, CNPY2 was chosen as a potential molecular target in liver carcinogenesis and subjected to comprehensive analysis.

Other protein expression changes detected by LC-Ms/Ms in mice HCAs and HCCs included elevation of several proteins with chaperon activity, binding to ER such as annexin A1 (ANEX1), heat shock 70kDa protein 5 (HSPA5), DEAD (Asp-Glu-Ala-Asp) box polypeptide 3, X-linked (DDX3X), tyrosine 3-monooxygenase/tryptophan 5-monooxygenase activation protein, beta (YWHAB), and zeta (YWHAZ). Furthermore, increase in proteins involved in oxidative stress responses and xenobiotic metabolism, such as isoenzymes of cytochrome P450 1A2 (CYP1A2), 2E1 (CYP2E1), 3A11 (CYP3A11), P450 (cytochrome) oxidoreductase (POR), carboxylesterase 1 (monocyte/macrophage serine esterase 1) (CES1), glutamate-ammonia ligase (glutamine synthetase) (GLUL), Y box binding protein 1 (YBX1) and cellular transporter apolipoprotein A1 (APOA1) was noted (Table 1). On the other hand, antioxidant system enzymes including catalase (CAT), superoxide dismutase 1 (SOD1), and enzymes of the urea cycle including carbamoyl-phosphate synthetase 1 (CPS1), ornithine carbamoyl transferase (OTC), ornithine aminotransferase (OAT) and arginase 1 (ARG1) were downregulated.

### 2.2. Altered Upstream Regulators and Signaling Pathways in Mice HCCs Predicted by Ingenuity Pathway Analysis (IPA)

**Figure 1:**
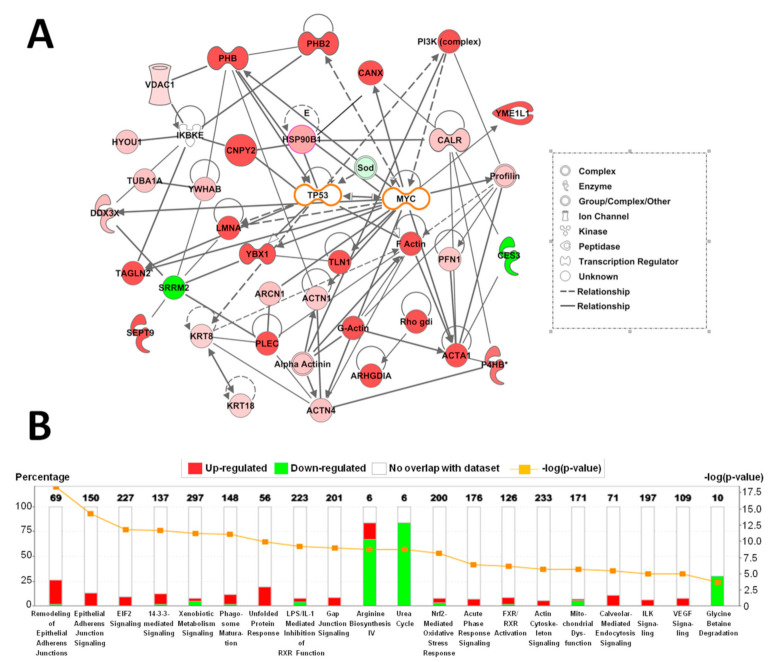
Ingenuity Pathway Analysis of alterations to protein expression in HCCs of C57Bl/6J mice induced by DEN treatment. (**A**) Networks involving differentially expressed proteins in mice HCCs. (**B**) Altered canonical pathways in HCCs from DEN-treated C57Bl/6J mice. Numbers of proteins with altered expression are represented as (−log (*p* values)).

Results of network and canonical pathway analyses by IPA are presented in Figure 1. IPA based on alterations of downstream proteins in C57Bl/6J mouse HCCs induced by DEN treatment demonstrated that altered networks could be in close relation with tumor suppressor TP53 (p53) and c-myc (MYC), but this needs further detailed investigation (Figure 1A). 

In addition, upstream regulator analysis in HCCs predicted activation of nuclear factor (erythroid-derived 2)-like 2 (Nrf2) and hepatocyte nuclear factor 4 (HNF4). Most differentially expressed proteins detected by QSTAR Elite Ms/Ms were targets for these transcriptional factors and contained binding sites. For instance, PHB, PHB2 and SEPT9 are known to be controlled by c-myc and Nrf2 [7]. On the other hand, PHB, PHB2, progesterone receptor membrane component 1 (PGRMC1) and downregulated enzymes of the urea cycle, might be regulated by HNF4 [7] (Table 1). 

Canonical pathway analysis by IPA demonstrated significant activation in remodeling of epithelial adherence junction signaling, gap junction signaling, EIF2- and 14-3-3-mediated signaling, the unfolded protein response, actin cytoskeleton signaling, ILK signaling and VEGF signaling in mouse liver HCCs (Figure 1B). Moreover, suppression of arginine, glycine biosynthesis and urea cycle pathways were predicted.

### 2.3. Immunohistochemical Assessment of CNPY2 and Related Proteins in Mice Livers

**Figure 2:**
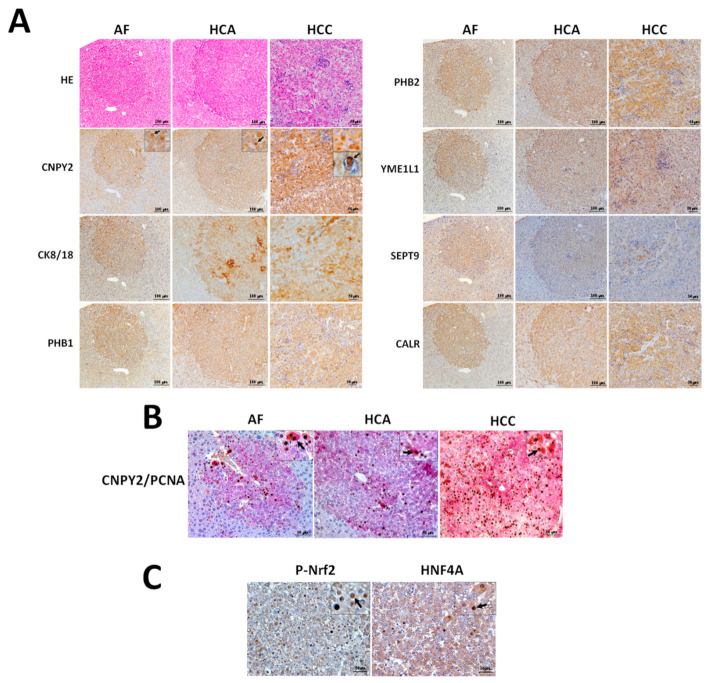
(**A**) Immunohistochemical detection of CNPY2, CK8/18, PHB1, PHB2, YME1L1, SEPT9 and CALR in liver specimens of C57Bl/6J mice at 27 (AF) and 38 (HCA and HCC) weeks after DEN initiation. (**B**) Overexpression of CNPY2 (red) and PCNA (brown/black) detected by double immunohistochemistry in AF, HCA and HCC of DEN-treated mice. Note significant overexpression of PCNA in the nuclei of CNPY2-positive cells comprising AF, HCA and HCC. (**C**) P-Nrf2 and HNF4A overexpression in HCCs of DEN-treated C57Bl/6J mice. Arrows: elevation of CNPY2, P-Nrf2 and HNF4A in the cytoplasm and nuclei of neoplastic cells. Note the coordinated staining pattern of CNPY2, CK8/18, PHB1, PHB2, YME1L1, SEPT9 and CALR in the mouse liver AF, HCAs and HCCs. Scale Bars: (**A**) 100 μm in AF and HCA and 50 μm in HCC; (**B**,**C**) 50 μm.

Differential expression of CNPY2 and other proteins detected by proteome analysis was verified in DEN-treated C57Bl/6J mice livers AF at week 27 and in HCAs and HCCs at week 38 by immunohistochemistry in serial sections (Figure 2A,B).

In support of the QSTAR Elite LC-Ms/Ms results, immunohistochemical examination demonstrated that preneoplastic lesions and tumors of DEN-initiated mice were positive for CNPY2, CK8/18, PHB1, PHB2, YME1L1, SEPT9 and CALR (Figure 2A). Elevation of CNPY2 in cytoplasmic endosomes was found in AF, HCA and HCC cells of DEN-treated mice, but not in the surrounding or control livers (Figure 2A). Interestingly, we also observed CNPY2-positive (CNPY2^+^) endosomes in the nuclei of HCC cells (Figure 2A). The function of CNPY2 in the nuclear endosomes of neoplastic lesions has to be further investigated. 

Double immunohistochemical assessment of cell proliferation marker PCNA and CNPY2 demonstrated significant elevation of PCNA-positive (PCNA^+^) cell nuclei numbers in CNPY2^+^ AF, HCAs and HCCs (Figure 2B). We further performed immunohistochemical detection of P-Nrf2 and HNF4, which activation was predicted on upstream regulator analysis by IPA, and in support, the results demonstrated elevation of P-Nrf2 and HNF4A in nuclei and cytoplasm HCC cells (Figure 2C).

### 2.4. In Vitro Functional Analysis of CNPY2

#### 2.4.1. Effects of CNPY2 Knockdown with siRNAs in Huh7 and HepG2 Human Liver Cancer Cells

**Figure 3:**
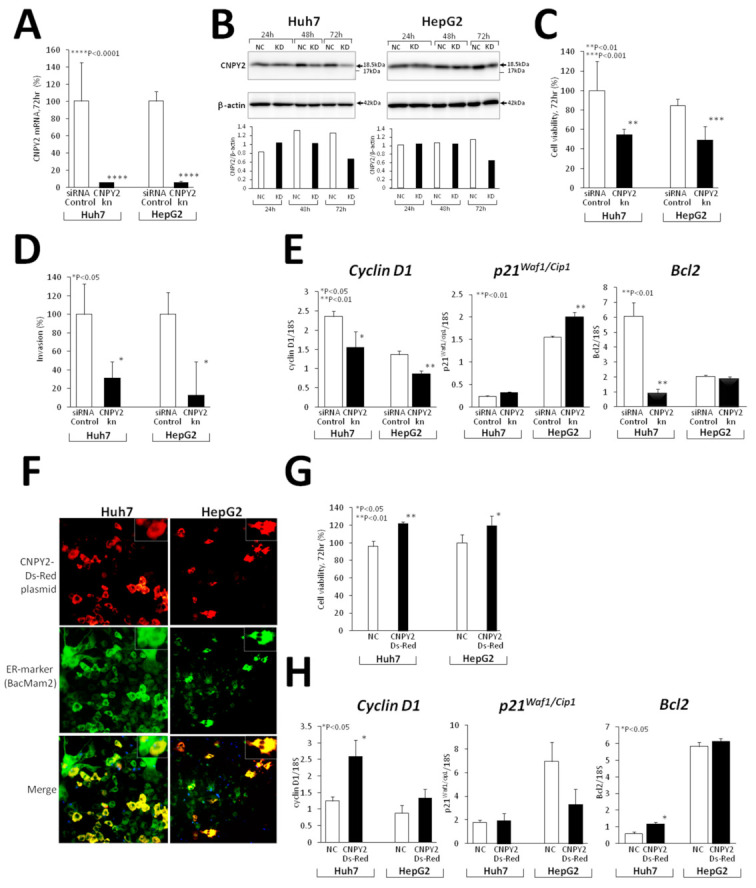
In vitro CNPY2 functional analysis in CNPY2kn Huh7 and HepG2 human liver cancer cell lines. (**A**–**E**) Experiments with knockdown of CNPY2 and (F–H) transfection of mCNPY2-Ds-Red plasmid vector in Huh7 and HepG2 cells. Downregulation of (**A**) CNPY2 mRNA and (**B**) protein levels in CNPY2kn cell lines detected by RT-PCR and Western blotting. (**C**) Suppression of cell viability and (**D**) invasion potential of CNPY2kn Huh7 and HepG2 cells. (**E**) Alterations of cyclin D1, p21^Waf1/Cip1^ and Bcl2 mRNA expression in CNPY2kn Huh7 and HepG2 cell lines detected by RT-PCR analysis. (**F**) Transfection of Huh7 and HepG2 cells with mCNPY2-Ds-Red plasmid and effects on cell survival and mRNA expression of cell cycle and apoptosis-related genes. Note the overexpression of CNPY2 in the endoplasmic reticulum (merged yellow color) in Huh7 and HepG2 cells, and increase in cell viability (**G**), cyclin D1 and Bcl2 mRNA expression (**H**) after the transfection of mCNPY2- Ds-Red in Huh7 and HepG2 cells.

Knockdown of CNPY2 was confirmed by RT-PCR and Western blot analyses. Thus, RT-PCR analysis demonstrated significant inhibition of CNPY2 mRNA expression after siRNA application as compared to controls in both Huh7 and HepG2 cell lines (Figure 3A). Western blot demonstrated a reduction in protein level of highly expressed 18.5 kDa processed variant of CNPY2 protein lacking ER signal peptide as compared with the siRNA control samples in both Huh7 and HepG2 cell lines transfected with CNPY2 siRNAs (Figure 3B and Figure S2). 

Furthermore, CNPY2 knockdown resulted in suppression of Huh7 and HepG2 cell viability and proliferation, as detected by WST8 assay (Figure 3C), as well as their invasion potential (Figure 3D), pointing out the important role of CNPY2 in cell survival and invasion processes. Next, we investigated effects of CNPY2 knockdown on cell cycle and apoptosis markers, and a significant decrease in cyclin D1 mRNA expression in knockdown Huh7 and HepG2 cell lines along with a trend for increase, and a significant elevation of p21^Waf1/Cip1^ mRNA in Huh7 and HepG2 cells, respectively, was found 72 h after siRNA application (Figure 3E). Moreover, expression of apoptosis inhibitor Bcl2 was significantly suppressed after CNPY2 knockdown in Huh7 cells.

Proteome analysis of CNPY2-knockdown (CNPY2kn) Huh7 and HepG2 cells was conducted using the QSTAR LC-Ms/Ms. Significantly underexpressed proteins and activated/inhibited upstream regulators detected in iTRAQ label and non-label proteome analyses in si-CNPY2kn-1 Huh7 and HepG2 cells are listed in Table 2 and Table S1. In CNPY2kn Huh7 and HepG2 cells, numerous cytoskeleton and ER stress-related proteins including CK8, CK18 and CK19, actin, beta-like 2 (ACTBL2), profilin 1 (PFN1), myristoylated alanine-rich protein kinase C substrate (MARCKS), MARCKS-like 1 (MARCKSL1), calumenin (CALU), calreticulin (CALR), calnexin, heat shock 70 kDa proteins 2, 9, and 5 were significantly downregulated (Table 2). In addition, expression of stress response-associated proteins, such as superoxide dismutase 2 (SOD2), epoxide hydrolase 1 (EPHX1), peroxiredoxin 1 (PRDX1) and 4 (PRDX4), Tu translation elongation factor (TUFM), DEAD (Asp-Glu-Ala-Asp) box polypeptide 39A (DDX39A) and famous HCC marker, alpha-fetoprotein (AFP), was decreased.

#### 2.4.2. mCNPY2-Ds-Red Plasmid Transfection in Human Liver Cancer Cells

To investigate the effect if CNPY2 transfection on cell viability and proliferation of Huh7 and HepG2 cells, we generated a mouse CNPY2 Ds-Red (mCNPY2-Ds-Red) plasmid, by cloning mouse CNPY2 mRNA containing ER signal peptide sequence into the Ds-Red plasmid vector. Staining with ER marker BacMam 2 (green color) and merging with mCNPY2-Ds-Red (red color) revealed that appreciable percentages of Huh7, HepG2 (>50%) were successfully transfected with mCNPY2-Ds-Red plasmid, and the transfected mCNPY2 protein (yellow color) was localized in the ER (Figure 3F). Next, mCNPY2-transfected Huh7 and HepG2 cell lines and negative controls were examined by WST8 assay, and the significant increases of cells viabilities were found after 72 h of incubation (Figure 3G), being indicative of the promoting effect of CNPY2 on cell survival.

In support of our previous results obtained in knockdown cell lines, RT-PCR analysis of mCNPY2-Ds-Red-transfected Huh7 and HepG2 cells demonstrated opposite changes of cell cycle and apoptosis markers (Figure 3H). Thus, the cell cycle regulator cyclin D1 mRNA expression was significantly elevated in both mCNPY2-Ds-Red-transfected Huh7 and HepG2 cells, a trend for decrease was found for p21^Waf1/Cip1^ mRNA in HepG2, while apoptosis inhibitor Bcl2 was significantly increased in Huh7 cells (Figure 3H). 

### 2.5. Expression of CNPY2 in HCV^+^ HCCs and Association with Clinicopathological Variables

**Figure 4:**
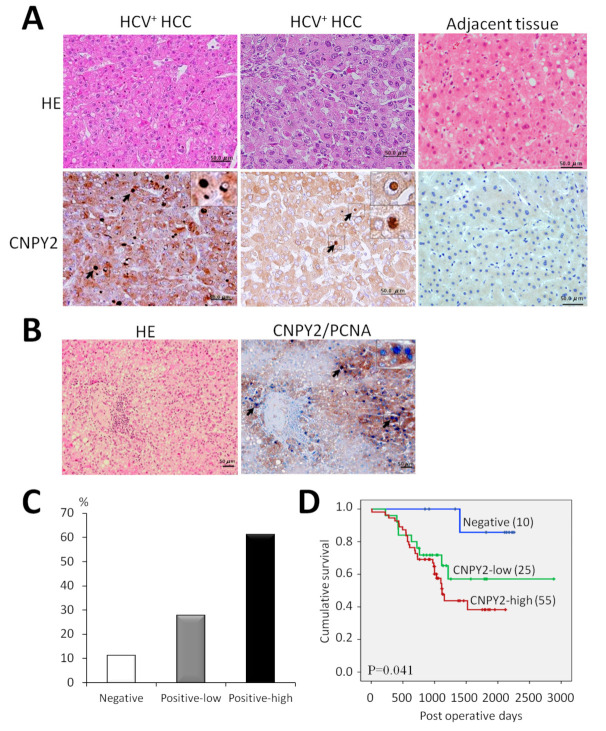
Representative images of CNPY2-positive human HCV^+^ HCCs and survival analysis. (**A**) Immunohistochemical findings for CNPY2 in human HCV^+^ HCCs and (**B**) Double staining for PCNA and CNPY2 in human HCC (brown: CNPY2-positive cytoplasm, blue: PCNA-positive nuclei). Scale bar: 50 μm. Note CNPY2 overexpression in the cytoplasm, cytoplasmic and nuclear endosomes of tumor cells, and significant elevation of PCNA-positive nuclei in CNPY2-positive area (arrows) in the tumor. (**C**) Percentages of CNPY2-positive and negative human HCV^+^ HCCs and (**D**) Cumulative survival curves in CNPY2-positive (high, low) and negative HCC patients.

To analyze the role of CNPY2 in human liver cancer, we next explored the relationship between CNPY2 expression with clinicopathological variables in HCV^+^ HCCs by immunohistochemistry (Figure 4, Table 3 and Table S2). CNPY2 elevation was predominantly observed in the cytoplasm of human HCC cells, likely in ER (Figure 4A). However, similar to mouse HCCs, CNPY2-positive nuclear endosomes also found in human tumor cells (Figure 4A). In support of our previous in vivo and in vitro results, double immunohistochemistry for CNPY2 and PCNA in human HCCs have demonstrated strong positive correlation of CNPY2 expression with cell proliferation (Figure 4B). Thus, CNPY2-positive areas (brown) of HCC contained high number of PCNA-positive nuclei (blue).

Quantification immunohistochemical analysis of 90 HCV^+^ HCC patients specimens demonstrated that CNPY2 was strongly elevated in 55 cases (61.1%; score 2+), weakly expressed in 25 (27.8%: score 1+) and negative in 10 (11.1%; score 0) cases (Figure 4C). Adjacent liver tissue was negative or slightly positive for CNPY2. Next, the association of CNPY2 expression and HCV^+^ HCC patients survival was investigated by performing the univariate survival analysis, according to the Kaplan–Meier method, and differences in cumulative survival were assessed with the log-rank test. Importantly, in HCV^+^ HCC patients, positive CNPY2 expression was associated with poorer cumulative (log-rank test; *p* = 0.041) survival, higher clinico-pathological stage (*p* = 0.016), pT factor (*p* = 0.016), poor histological tumor differentiation (*p* = 0.035) and venous invasion (*p* = 0.038), as compared with its negative expression (Figure 4D and Table 3). No statistically significant associations were observed between increased CNPY2 expression (scores; 0, 1+, 2+) and gender, smoking, drinking, past history of diabetes, stage of cirrhosis, tumor size, established prognostic factors of im, pB and pM status of HCV^+^ HCC patients (Table 3). No lymph node metastases (pN status) were observed in HCV^+^ HCC patients. 

On univariate analysis, CNPY2 overexpression was significantly associated with poor prognosis in 90 HCV^+^ HCC patients (hazard ratio (HR), 7.117; 95% confidence interval (CI), 0.971–52.18, *p* = 0.049). From these results, CNPY2 overexpression was thus indicated to be an independent prognostic factor for HCV^+^ HCC patients survival.

## 3. Discussion

In the present study, the role of CNPY2 was investigated in mouse preneoplastic and neoplastic lesions and human virus-associated hepatocarcinogenesis. The results of proteome and immunohistochemical analyses demonstrated marked elevation of CNPY2 in DEN-treated mouse livers, AF, HCAs and HCCs. It was apparent that the upregulation in liver lesions correlated with significant elevation of cellular proliferation indicative of an important role for the protein in the early stages of mouse hepatocarcinogenesis. Furthermore, we provided the first experimental evidence for coordinated elevation of CNPY2, CK8/18, SEPT9, CALR, PHB1, PHB2 and YME1L1, in mouse liver preneoplastic lesions and tumors. Nrf2 and HNF4A transcriptional factors were elevated in mouse HCCs being active in the last stages of hepatocarcinogenesis. 

CNPY2 encodes a predicted 182- and 162-amino acid type II transmembrane protein with apparent molecular weights of 20.65 and 18.54 kDa (the intact and processed variants with and without the ER signal peptide, respectively). CNPY2 is localized in the ER, plasma membrane, intracellular space and perinuclear region of cells, and is known to exhibit positive expression in the brain, kidney, lung, heart, skeletal muscle, fibroblasts, adult placenta, heart, lung, liver and pancreas, uteri and was also proved detectable in mouse blood [11]. CNPY2 has been shown to participate in outgrowth of neurites by increasing MRLC phosphorylation [8], which is a key regulatory mechanism in the control of myosin activity and cell motility, and to enhance cell spreading of fibroblasts and migration of rat C6 glioma cells [9].

It is of major interest that in vitro functional analysis in human liver cancer cell lines demonstrated that knockdown of CNPY2 inhibited cell growth, survival and proliferation due to suppression of cyclin D1, induction of cyclin-dependent kinase inhibitor p21^Waf1/cip1^ and downregulation of the apoptosis inhibitor Bcl2 [12]. On the contrary, transfection with a CNPY2 vector plasmid and consequent expression in ER of Huh7 and HepG2 cells enhanced their survival and exerted opposite effects on cyclin D1, p21^Waf1/cip1^ and Bcl2. Overexpression of cyclin D1 in various human cancers has been regarded as a key mechanism underlying tumor growth, angiogenesis, progression, and metastasis [13,14]. Furthermore, upregulation of cyclin D1 in HCCs has been noted to be associated with aggressive tumor forms [15]. Suppression of cyclin D1 in Huh7 and HepG2 human liver cancer cell lines by anti-tumor agents was earlier suggested to block cyclin D1 turnover [16,17]. 

Importantly, CNPY2 knockdown also strongly suppressed invasion activity of Huh7 and HepG2 cells. In addition, inhibition of numerous proteins involved in cytoskeleton organization including the system of intermediate and thin filaments (actomyosin complex) and ER stress were observed in CNPY2kn liver cancer cells. These data are in line with previous report suggesting of CNPY2 to be HIF-1alpha-regulated, secreted angiogenic growth factor that promotes smooth muscle cell SMC migration, proliferation, and tissue revascularization [18], to be regulated by thioguanine nucleotide and to bind to MYLIP, MARCH8, TNIK, PINX1, IKBKE, and MIR207 [8,19]. 

In line with our results, critical roles of CNPY2 in ER stress and unfolded protein response-related diseases such as metabolic disorders, inflammation and cancer through regulation of protein kinase R-like ER kinase (PERK)-C/EBP homologous protein (CHOP) pathway have been proposed [10]. As an ER luminal protein CNPY2 has been shown to be released from grp78 upon ER stress and to be transcriptionally upregulated by CHOP signaling [10]. Therefore, we propose here that induction of ER stress and unfolded protein response by CNPY2 due to its ability to suppress proteolysis [10], and stabilize actomyosin complexes may play roles and promote the growth of podia during invasion and metastasis of tumor cells (Figure S3).

Interestingly, in the present study, CNPY2-positive endosomes were observed not only in the cytoplasm, but also in the nuclei of HCC cells indicating that CNPY2 function in tumorigenesis might not be limited to ER. Recently, certain endocytic proteins involved in endocytosis were reported to undergo nucleocytoplasmic shuttling and/or interact with nuclear molecules involved in transcription or chromatin remodeling in response to extracellular signals [20]. 

To the best of our knowledge, this study is first to demonstrate CNPY2 overexpression in high grade HCV^+^ HCCs confirmed by cytoplasmic staining patterns. Importantly, positive CNPY2 expression was associated with poorer overall survival, vessel invasion, poor histological tumor differentiation, higher clinico-pathological stage and pT factor in HCV^+^ HCC cases, as compared with negative expression, indicating that CNPY2 is likely to play an important role in cancer progression. This is in line with the recent finding that CNPY2 may be a valuable prognostic indicator for several other types of neoplasms including the colorectal cancer, renal cell carcinoma, lung and esophageal squamous cell carcinoma [12,21,22,23,24]. CNPY2 potential as a novel molecular target in hepatocarcinogenesis, and as an independent prognostic factor for different types of human liver cancer warrants further exploration.

## 4. Materials and Methods

### 4.1. Chemicals

Reagents were purchased from Sigma (St. Louis, MO, USA) or Wako Pure Chemical Industries (Osaka, Japan). Zwittergent was obtained from CALBIOCHEM (EMD Biosciences, La Jolla, CA, USA). All standards and reagents were of analytical grade. 

### 4.2. Institutional Review Board Approval

Animal experimentation was performed in accordance with the Guidelines of Public Health Service Policy on the Humane Use and Care of Laboratory Animals, and the National Institute of Health and further approved (06024) by the Ethics Committee of the Institutional Animal Care and Use Committee of Osaka City University Graduate School of Medicine, Osaka, Japan. Experiments with human samples were approved (2263) by the Osaka City University Graduate School of Medicine Ethics Committee. 

### 4.3. Liver Tissue from In Vivo Experiment

Serial paraffin sections were prepared from preneoplastic lesions and tumor containing livers of mice from our previous experiment [6]. In short, twenty-six 2-week-old male C57Bl/6J mice were injected a single intraperitoneal dose of N-nitrosodiethylamine (DEN) (10 mg/kg b.w., i.p.) dissolved in saline to initiate hepatocarcinogenesis. A further 12 mice in a control group were administered an i.p. injection of saline. Livers and liver tumors were isolated at 27 and 38 weeks after the carcinogen treatment. At sacrifice, livers were immediately excised and macroscopically examined so that tumors and normal-appearing liver tissue could be sectioned and separate portions fixed in 10% phosphate-buffered formalin for proteome, histopathological and immunohistochemical analyses [6]. The method developed for proteome analysis of microdissected preneoplastic lesions and tumors from formalin-fixed and paraffin-embedded (FFPE) sections stained in hematoxylin solution is described below and demonstrated in Figure S4.

### 4.4. Laser Microdissection

For QSTAR Elite LC-Ms/Ms analysis, basophilic AF (27 week-point), HCAs, HCCs (38 week-point), peri-tumoral (surrounding) or normal-appearing liver areas from the livers of C57Bl/6J mice in DEN initiation and vehicle control groups were microdissected from hematoxylin-stained FFPE liver sections using the PALM MicroBeam System with Laser Microdissection and Pressure Catapulting technology (LMPC) (P.A.L.M. Microlaser Technologies AG, Meiwa Shoji Co., Ltd., Germany) according to previously reported protocols [7,25,26]. Each LCM cap transferred about 3000 cells (~5,000,000 μm^2^ area) for tumors and 1000 cells (1,666,667 μm^2^) for AF samples. To each LCM cap, 5 μL of 20 mM Tris-HCl buffer, pH 8, with 0.002% Zwittergent lysis buffer were added and samples were suspended. 

### 4.5. Protein Identification in Microdissected Samples by QSTAR Elite LC-Ms/Ms

Proteome analysis was performed with the DiNa-AI nano LC System (KYA Technologies, Tokyo, Japan) coupled to a QSTAR Elite hybrid mass spectrometer (AB Sciex, Concord, ON, Canada) through a NanoSpray ion source (AB Sciex, Concord, ON, Canada) as previously described [7]. Samples from microdissected AF, tumors and normal-appearing liver tissues of mice in DEN initiation and vehicle control groups, respectively, were treated with heat at 90 °C for 90 min (spin down every 20 min), digested with trypsin at 37 °C for 16–18 h and centrifuged at 10,500 rpm for 1 min. Protein concentrations were measured with a BCA Protein Assay Kit (Pierce, Rockford, IL, USA). AF and normal liver samples at 27 week-point contained 5 µg aliquots of protein. HCAs, HCCs and normal liver at 38 week-point contained 20 µg aliquots. For the quantitative LC-Ms/Ms analysis, samples were labeled with 4-plex iTRAQ reagents as described below. First set: 114 iTRAQ label, microdissected AF in DEN-treated mice (27 weeks); 115 iTRAQ label, normal-appearing liver area in vehicle control group (27 weeks). Second set: 114 iTRAQ label, microdissected HCAs in DEN-treated mice (38 weeks); 115 iTRAQ label, microdissected HCCs in DEN-treated mice (38 weeks); 116 iTRAQ label, peri-tumoral liver area in DEN-treated mice (38 weeks); 117 iTRAQ label, normal-appearing liver area in vehicle control group (38 weeks). Protein ratios between iTRAQ labeled samples detected by ProteinPilot software with a *p*-value less than 0.05 were considered reliable. To identify total protein spectra, in the non-quantitative (no label) analysis, the same non-labeled samples were subjected to LC-Ms/Ms according to standard procedures [27]. In no label analysis, we picked up proteins overexpressed (detected only in experimental group) or underexpressed (detected only in the control group) in mice livers.

### 4.6. Ingenuity Pathway (IPA) Analysis

To identify networks of interacting proteins, functional groups and pathways, the Ingenuity program (Ingenuity Systems, Mountain View, CA, USA) was employed. To identify common transcriptional factors that could be driving protein expression changes, the focus was on upstream regulators measured by the z-score, with values >2 (activation), or <−2 (inhibition) considered significant. Canonical pathway analysis included alterations of pathways with z-scores above 1.5. The basis for z-score predictions are relationships in the molecular pathways (networks) which reflect experimentally observed protein expression or transcription events.

### 4.7. Immunohistochemistry and Scoring

Immunohistochemical examination was performed to confirm the results and to detect the localization of target proteins using standard protocols as recently described [6,7]. FFPE sections of mouse normal liver and samples with preneoplastic and neoplastic lesions were stained using standard immunohistochemical methods. Rabbit polyclonal antibodies against canopy 2 homolog (CNPY2) (1:300, 14635-1-AP, ProteinTech Group, Inc., Rosemont, IL, USA), YME1-like-1 (YME1L1) (1:200, 11510-1-AP, ProteinTech Group, Inc., Rosemont, IL, USA), HNF4A (K307) (1:100, BS2983, Bioworld Technology Inc., St. Louis Park, MN, USA), guinea pig polyclonal antibodies against CK8 and 18 (CK8/18) (dilution 1:600, Progen Biotechnik, Germany) and rabbit monoclonal antibodies against p-Nrf2 (S40) (1:300, ab76026, Abcam, Tokyo, Japan) were employed for validation of the proteome and IPA analyses results. Furthermore, we generated new monoclonal rat PHB1, PHB2 and SEPT9 antibodies, using the lymph node method (all from Risk Assessment and Research Inc., Japan) [28]. With formalin-fixed sections microwave antigen retrieval was performed in citrate buffer (pH 6.0). 3,3′-diaminobenzidine tetrahydrochloride (DAB) was used for antigen visualization. In double immunohistochemistry for CNPY2 and PCNA, CNPY2 was visualized either with DAB (brown) or alkaline phosphatase (red) solutions, while PCNA was stained with DAB (brown/black) or alkaline phosphatase (blue) using methods previously described [5,6]. PCNA mouse monoclonal antibodies (1:500, M0879, Dako, Japan) were used. All immunohistochemical procedures were optimized by testing negative controls and antigen retrieval methods. Immunohistochemically stained slides were examined by at least two pathologists and graded as score 0 (negative), 1+ (low) and 2+ (high), as follows: score 0, negative staining in all cells; score 1+, positive staining in <50% of cells; score 2+, positive staining in >50% cells.

### 4.8. Patients and Tissue Samples

Primary tumor tissues and matched adjacent non-tumor samples were obtained from 90 HCV-positive patients with histologically proven primary HCCs who underwent surgical resection at Osaka City University Hospital (Osaka, Japan) from January 2006 to December 2016. Patient clinical features such as age, gender, smoking history, tumor volume and tumor differentiation were obtained from medical records. There were 62 male and 28 female HCV^+^ HCC patients, with a median age of 72 years at the time of surgery. Pathological diagnoses and staging were performed by at least two pathologists from the Pathology Departments in our Medical School according to the criteria of the general guidelines for primary liver cancer of the American Joint Committee on Cancer/International Union Against Cancer staging systems [29] and the Liver Cancer Study Group of Japan in accordance with the Japanese classification of HCC [30]. Fifty-four of the 90 HCV^+^ HCC patients were diagnosed with recurrence. To assess alterations of CNPY2 expression, we analyzed expression levels in HCCs and adjacent liver areas. Overall survival was measured from the date of diagnosis until death from any cause or the date of last follow up. Clinicopathological data, including age, sex, tumor size, pathological T (pT), M (pM) and B (pB) factors, intrahepatic metastasis (im), vessel invasion, aspartate transaminase (AST) and alanine transaminase (ALT) were recorded. 

### 4.9. In Vitro Experiments

#### 4.9.1. Cell Lines and Culture Conditions

The Huh7 and HepG2 human HCC cell lines were purchased from the Japanese Collection of Research Bioresources (Osaka, Japan) and routinely maintained in Dulbecco’s modified Eagle’s medium (DMEM) and Minimum Essential Medium (MEM), respectively (Invitrogen, Carlsbad, CA, USA), supplemented with 1% penicillin/streptomycin and 10% fetal bovine serum (FBS; Invitrogen). All cells were incubated at 37 °C in a 5% CO_2_ air-humidified atmosphere.

#### 4.9.2. siRNA Knockdown of CNPY2 in Human Liver Cancer Cells

CNPY2 expression was transiently knocked down in Huh7 and HepG2 human liver cancer cells using Lipofectamine RNAiMAX (Invitrogen, Carlsbad, CA, USA) according to the manufacturer’s instructions. CNPY2-specific siRNA (Silencer Select siRNA Cat.No.4392420; CNPY2 IDs: s20206 (A: siRNA-1), s20207 (B: siRNA-2) and s20208 (C: siRNA-3)) were obtained from Thermo Fisher Scientific K.K. (Tokyo, Japan). Non-targeting control siRNA (Silencer Select, Cat.No.: 4390843, Ambion, Tokyo, Japan) was obtained from Life Technologies (Tokyo, Japan). Huh7 and HepG2 cells (5 × 10^4^/well) were transiently transfected with 6.7 nM CNPY2 siRNAs or control siRNA in 24-well plates. After 24 h, CNPY2kn cells were trypsinized and used for Western blot and Q-RT-PCR analysis. Knockdown with s20206 si-RNA (si-RNA-1) demonstrated the best results in all in vitro assays.

#### 4.9.3. Generation of the Mouse CNPY2 Containing Vector

Mouse CNPY2 cDNA featuring deletion of 4 amino acids (HDEL) at the C-terminus was produced by RT-PCR reaction and subcloned into the pcDNA3.1 plasmid (Invitrogen, Carlsbad, CA, USA) together with the DS-Red sequence from the DsRed2-N1 vector (54493, Addgene, Florida State University, Tallahassee, FL, USA) and HDEL at the C-terminus according to the manufacturer’s instructions.

#### 4.9.4. Transfection of CNPY2-Containing Vector in Huh7 and HepG2 Liver Cancer Cell Lines

CNPY2 vector expression was transiently transfected in Huh7 and HepG2 cells using Lipofectamine 3000 reagent (Invitrogen, Carlsbad, CA, USA) according to the manufacturer’s instructions. Huh7 and HepG2 cells (3 × 10^4^/well) were transiently transfected with 2 µg mCNPY2-Ds-Red vector or control DNA in 24-well plates according to the manufacturer’s protocol. After 24 h, cells were stained with BacMam2 (ThermoFisher Scientific K.K., Tokyo, Japan), a marker of ER and DAPI as prescribed in the supplied protocol.

Huh7 and HepG2 human liver cancer cells were transfected with the mouse CNPY2 vector as described above, and changes in survival and mRNA expression of cyclin D1, p21^Waf1/cip1^ and Bcl2 were evaluated using the WST8 assay and RT-PCR analysis using protocols described below. Fluorescent detection of the ER marker BacMam2 was performed in all cells transfected with the mouse CNPY2 vector mCNPY2-Ds-Red, and negative controls.

#### 4.9.5. WST-8 Assay

In knockdown experiment, Huh7 and HepG2 cells (1 × 10^4^/well) were transiently transfected with 6.7 nM CNPY2 siRNAs or control siRNA in 96-well plates as described above, and used 24 h later in WST-8 assay. In plasmid transfection experiment, Huh7, HepG2 (1 × 10^4^/well) were transfected with the mCNPY2-Ds-Red vector (1 µg DNA/well) or the control DNA. In both experiments, 24, 48, 72 and 96 (Huh7, HepG2) h after the transfection, cells were washed with warm media, and 10 μL of filtered solution of water-soluble tetrazolium salt, WST-8, from the Cell Counting Kit-8 (Dojindo Molecular technologies Inc., Kumamoto, Japan) was added to each well, followed by 1 hr incubation at 37 °C. Absorbance of the converted dye was measured at a wavelength of 450 nm with background subtraction at 600 nm on a microplate reader (Bio-Rad, Tokyo, Japan).

#### 4.9.6. Invasion Assay

In vitro cell invasion assays were performed for CNPY2 knockdown and control Huh7 and HepG2 cells in triplicate as previously described [31]. Tumor cells that migrated through transwell inserts with a uniform layer of BD Matrigel basement membrane matrix (BD Biosciences, Bedford, MA, USA) were assessed according to the manufacturer’s instructions. Briefly, transfectants (Huh7: 2.5 × 10^5^ cells; HepG2: 4 × 10^5^ cells) were seeded in 500 μL of serum-free medium in the upper chamber, whereas the lower chamber was loaded with medium containing 10% FBS. After 22 h, the non-invading cells were removed with a cotton swab, the invading cells were stained with 1% toluidine blue and counted under a light microscope.

#### 4.9.7. Real-Time Quantitative PCR

Total RNA was extracted from human liver cancer cells using an RNeasy Mini Kit (QIAGEN, Carlsbad, CA, USA). Reverse transcription of 1 μg of total RNA was performed with Oligo-dT primers. Real-time quantitative PCR was performed on an ABI Prism 7000 (Applied Biosystems, Foster City, CA, USA), using TaqMan probes and primer sets from TaqMan Gene Expression Assays (4331182) (Thermo Fisher Scientific, Japan) for the analysis of mRNA expression of CNPY2 (NM_019953.1), cyclin D1 (CCND1: Hs00277039_m1), p21^Waf1/cip1^ (CDKN1A: Hs00355782_m1) and Bcl2 (Hs00608023_m1) [32]. Results were expressed relative to the number of eukaryotic 18S rRNA (4319413E) (Applied biosystems, Japan) transcripts used as an internal control. All measurements were performed in triplicate.

#### 4.9.8. Protein Extraction and Western Blot Analysis

Protein extraction from cultivated cells and Western blot analyses were performed as previously described [31]. In brief, whole cell lysates were collected using a cell scraper and resuspended in CelLytic MT (Sigma, St. Louis, MO, USA) with a protease inhibitor. Cells were lysed in buffer containing 25 mM Tris (pH 7.4), 100 mM NaCl and 1% Tween-20. The amount of total protein was determined using a BCA protein assay kit (Pierce). Protein (10 μg aliquots) was loaded onto 12.5% SDS-polyacrylamide gels and separated by SDS-PAGE. The resolved proteins were electrophoretically transferred to polyvinylidene fluoride (PVDF) membranes (Bio-Rad, Inc., Hercules, CA, USA) and blocked with 5% skimmed milk in TBS buffer containing 0.1% Tween-20 for 1 h at room temperature. The membranes were then probed with primary rabbit polyclonal antibodies against CNPY2 (1:1000, HPA038465, ATLAS Antibodies, Stockholm, Sweden) or β-actin (1:100,000, ab49900, Abcam, Japan) for 1 h at room temperature. After washing, membranes were incubated for 1 h at room temperature and linked with HRP-conjugated secondary antibody (sc-2004, 1:10,000; Santa Cruz Biotechnology, Santa Cruz, CA, USA). Immunoreactive bands were detected using the ECL Plus Western blotting system (GE Healthcare, Piscataway, NY, USA) and FUSION-Chemiluminescence Imaging System (M&S Instruments Inc, Osaka, Japan). WIDE-VIEWTM Prestained Protein Size Marker Ⅲ was applied for molecular weight analysis (Fujifilm Wako Pure Chemical Corporation, Osaka, Japan).

#### 4.9.9. QSTAR LC-Ms/Ms and Ingenuity Pathway Analysis (IPA) of CNPY2kn Huh7 and HepG2 Cells

Proteome analysis in CNPY2kn-1 Huh7 and HepG2 human liver cancer cells (20 μg protein) was performed using the DiNa-AI nano LC System (KYA Technologies, Tokyo, Japan) coupled to a QSTAR Elite hybrid mass spectrometer (AB Sciex, Concord, ON, Canada) through a NanoSpray ion source (AB Sciex, Concord, ON, Canada) as previously reported [7]. Samples were labeled as follows: 114: CNPY2kn-1 Huh7/HepG2; 115: negative control Huh7/HepG2 cell lysates. IPA (Ingenuity Systems, Mountain View, CA, USA) was employed for analysis of protein molecular functions and altered up-stream regulators. 

### 4.10. Statistical Analysis

The StatLight–2000(C) program (Yukms corp., Yokohama, Kanagawa, Japan) was used for the statistical analysis of the results of in vivo and in vitro experiments. ProteinPilot™ 2.0 Software was employed for the statistical analysis of the quantitative changes of protein expression in mice HCCs and CNPY2kn Huh7 and HepG2 human liver cancer cells. First, the F test was used for the evaluation of the significance of differences between mean values. If non-homogeneous, the results were then analyzed with the Welch test, and if homogenous, with the Student’s t-test (two-sided) using SPSS statistics version 19.0. (SPSS Inc., Chicago, IL, USA). Statistical significance of associations between CNPY2 staining and various clinicopathological variables was evaluated using chi-square and Fisher’s tests. Survival curves were calculated from the day of surgery to relapse or death or to the last follow-up using the Kaplan–Meier method, and differences in overall survival curves were assessed with the Log rank test. In all applied analyses, *p*-values <0.05 were considered statistically significant. Univariate Cox proportional hazard model analysis was performed to calculate hazard ratios (HRs) and to determine associations between CNPY2 overexpression and disease-specific mortality.

## 5. Conclusions

In conclusion, the present results indicate that CNPY2 may become a novel promising molecular and theraupetic target in liver cancer, with involvement in the processes of ER stress, cell survival, proliferation and invasion of tumor cells.

## Figures and Tables

**Table 1:** Differentially expressed proteins in mouse liver AF, HCAs and HCCs.

Protein	Liver	AF	HCA	HCC
canopy 2 homolog (zebrafish) (CNPY2)	-	↑	↑	↑
heat shock 70kDa protein 5 (glucose-regulated protein, 78kDa) (HSPA5)	1.20	-	-	2.73
heat shock protein 90kDa beta (Grp94), member 1 (HSP90B1)	-	-	-	2.19
fission 1 (mitochondrial outer membrane) homolog (FIS1)	-	-	-	2.05
calreticulin (CALR)	-	↑	↑	↑
prohibitin 1 (PHB1)	-	↑	↑	↑
prohibitin 2 (PHB2)	-	↑	↑	↑
YME1-like 1 (S. cerevisiae) (YME1L1)	-	↑	↑	↑
cytokeratin 18 (CK18)	1.27	2.1	1.49	1.52
cytokeratin 8 (CK8)	1.21	2.5	1.54	1.57
fibronectin 1 (FN1)	-	-	-	↑
actin, alpha 1, skeletal muscle (ACTA1)	-	-	-	↑
actin, gamma 2, smooth muscle, enteric (ACTG2)	-	-	-	↑
Rho-associated, coiled-coil containing protein kinase 2 (ROCK2)	-	-	-	↑
myosin, heavy chain 9, non-muscle (MYH9)	-	-	-	↑
septin 9 (SEPT9)	-	↑	↑	↑
talin 1 (TLN1)	-	-	-	↑
annexin A1 (ANXA1)	-	-	-	↑
S100 calcium binding protein A8 (S100A8)	-	-	-	3.46
plectin (PLEC)	-	-	-	↑
Rho GDP dissociation inhibitor (GDI) alpha (ARHGDIA)	-	-	-	↑
aldehyde dehydrogenase 3 family, member A2 (ALDH3A2)	-	-	2.79	2.05
flavin containing monooxygenase 5 (FMO5)	-	-	1.73	2.35
superoxide dismutase 1, soluble (SOD1)	-	-	-	0.61
catalase (CAT)	-	-	-	0.79
protein disulfide isomerase family A, member 6 (PDIA6)	1.10	-	-	3.18
prolyl 4-hydroxylase, beta polypeptide (P4HB)	-	-	-	2.08
cytochrome P450, family 1, subfamily A, polypeptide 2 (CYP1A2)	-	-	2.37	1.39
cytochrome P450, family 2, subfamily E, polypeptide 1 (CYP2E1)	1.32	-	1.51	2.98
cytochrome P450, family 3, subfamily A, polypeptide 11 (CYP3A11)	-	-	-	2.83
glutathione S-transferase mu 2 (GSTM2)	-	-	4.50	3.50
glutathione S-transferase theta 1 (GSTT1)	-	-	-	↑
carboxylesterase 1 (monocyte/macrophage serine esterase 1) (CES1)	1.10	2.88	1.60	2.74
UDP-glucose pyrophosphorylase 2 (UGP2)	-	-	-	2.63
glutamate-ammonia ligase (glutamine synthetase) (GLUL(GS))	-	2.34	9.73	5.61
ornithine aminotransferase (OAT)	-	-	-	0.28
arginase, liver (ARG1)	-	-	-	0.64
argininosuccinate lyase (ASL)	1.07	-	0.23	1.48
argininosuccinate synthetase 1 (ASS1)	0.77	-	0.14	2.46
carbamoyl-phosphate synthase 1, mitochondrial (CPS1)	1.12	-	-	0.38
ornithine carbamoyltransferase (OTC)	1.34	-	-	0.67
alpha-2-HS-glycoprotein (AHSG)	-	-	-	↑
Y box binding protein 1 (YBX1)	-	↑	↑	↑
apolipoprotein A-I (APOA1)	1.35	↑	↑	8.73
progesterone receptor membrane component 1 (PGRMC1)	-	-	-	2.57
DEAD (Asp-Glu-Ala-Asp) box polypeptide 3, X-linked (DDX3X)	-	-	-	2.03

Data are fold of protein expression vs. non-treated liver of C57Bl/6J mice. ↑: overexpression detected in no-label analysis (no expression observed in non-treated controls); -, no change.

**Table 2:** Differentially expressed proteins in Huh7 and HepG2 human liver cancer cells after the knockdown of CNPY2.

Name (Symbol)	ID (GI no.)	CNPY2kn Huh7/HepG2
ER and Oxidative Stress Response		
calnexin (CANX)	543920	−2.11/−1.88
calreticulin (CALR)	117501	↓/↓
calumenin (CALU)	5921197	↓/↓
heat shock 70kDa protein 2 (HSPA2)	1708307	↓/↓
heat shock 70kDa protein 9 (mortalin) (HSPA9)	21264428	↓/↓
heat shock 70kDa protein 5 (glucose-regulated protein, 78kDa) (HSPA5)	14916999	−2.10/−3.10
Tu translation elongation factor, mitoch. (TUFM)	1706611	↓/↓
superoxide dismutase 2, mitochondrial (SOD2)	134665	↓/↓
epoxide hydrolase 1, microsomal (xenobiotic) (EPHX1)	123926	−2.21/−1.73
peroxiredoxin 1 (PRDX1)	548453	↓/↓
peroxiredoxin 4 (PRDX4)	3024727	−2.12/−1.53
Cytoskeleton organization		
cytokeratin 8 (CK8)	90110027	−2.15/−2.77
cytokeratin 18 (CK18)	125083	↓/↓
cytokeratin 19 (CK19)	311033484	↓/↓
actin, beta-like 2 (ACTBL2)	172046825	−2.26/−2.92
myristoylated alanine-rich protein kinase C substrate (MARCKS)	76803798	↓/↓
MARCKS-like 1 (MARCKSL1)	1346576	↓/↓
profilin 1 (PFN1)	130979	↓/↓
cofilin 1 (non-muscle)(CFL1)	116848	↓/↓
tropomyosin 4 (TPM4)	530415128	↓/↓
Others		
DEAD (Asp-Glu-Ala-Asp) box polypeptide 39A (DDX39A)	61212932	−2.18/−1.52
alpha-fetoprotein (AFP)	120042	−2.77/−2.43

Data are mean fold changes of all differentially expressed proteins in CNPY2kn Huh7 and HepG2 cell lines subjected to analysis. ↓: Decreased protein levels detected in no-label analysis (no expression observed in CNPY2kn cell lines).

**Table 3:** Correlation between CNPY2 expression and clinicopathological variables of HCV^+^ HCC patients.

Factors	CNPY2		
	(+) (*n* = 80)	(−) (*n* = 10)	*p*
Age			0.260
>65	59(74%)	9(90%)	
≤65	21(26%)	1(10%)	
Gender			0.421
Male	54(67%)	8(80%)	
Female	26 (33%)	2(20%)	
Smoking			0.330
Smoker	43(54%)	7(70%)	
Non-smoker	37(46%)	3(30%)	
Drinking			0.171
Drinker	30(37%)	6(60%)	
Non-drinker	50(63%)	4(40%)	
Diabetes			0.530
(+)	17(21%)	3(30%)	
(−)	63(79%)	7(70%)	
Cirrhosis			0.331
Stage 1&2	35(44%)	6(60%)	
Stage 3&4	45(56%)	4(40%)	
Tumor size			0.256
<20 mm^3^	46(58%)	7(78%)	
≥20 mm^3^	33(42%)	2(22%)	
AST			0.496
13–33	20(25%)	3(30%)	
>34 ng/mL	60(75%)	7(70%)	
ALT			0.225
6–27	54(68%)	5(50%)	
>28 ng/mL	26(32%)	5(50%)	
pT			0.016
T1	19(24%)	6(60%)	
T2–T4	61(76%)	4(40%)	
pM			0.722
(+)	79(99%)	0(0%)	
(−)	1(1%)	10(100%)	
pB			0.722
(+)	79(99%)	0(0%)	
(−)	1(1%)	10(100%)	
Venous invasion			0.038
(+)	55(69%)	0(0%)	
(−)	25(31%)	10(100%)	
Differentiation ^a^			0.035
Well	5(6%)	3(30%)	
Moderate	33(41%)	2(20%)	
Poor	42(53%)	5(50%)	
Clinical Stage ^b^			0.016
I	19(24%)	6(60%)	
II	40(50%)	1(10%)	
III	18(22%)	3(30%)	
IV	3(4%)	0(0%)	
im			0.681
(+)	12(15%)	2(20%)	
(−)	68(85%)	8(80%)	

Pearson’s Chi square test; pT: pathological T factor; pM: pathological M factor; pB, pathological B factor; im, intrahepatic metastasis; ^a^ Well and Moderately vs. Poorly differentiated; ^b^ Stage I vs. Stages II&III&IV.

## Data Availability

The data obtained in this study are publically available in the article and supplementary materials.

## Supplementary Materials

The following are available online at https://www.mdpi.com/article/10.3390/cancers13143613/s1, Figure S1: Overexpression of CNPY2 in HCCs of DEN-treated C57Bl/6J mice detected by Western blot analysis, Note very low expression of CNPY2 in non-treated mice livers, small increase in non-healthy surrounding livers and high expression in HCCs. N: normal liver; S: surrounding (peri-tumoral) liver, Figure S2: CNPY2 knockdown in Huh7 and HepG2 cells detected by Western Blot analysis, Figure S3: A proposed effect of CNPY2 knockdown on ER stress, actomyosin-ECM system and invasion activity of liver cancer cells. N: nuclear; ER: endoplasmic reticulum, ECM: extracellular matrix, Figure S4: Developed method for sensitive proteome analysis in laser-microdissected samples of altered foci and liver tumors of DEN-treated and control C57Bl/6J mice, Table S1: Up-stream regulator analysis by IPA in CNPY2kn Huh7/HepG2 cell lines, Table S2: Patient clinicopathological characteristics, tumor characteristics, and treatment characteristics for HCV^+^ HCC patients (*N* = 90).
