# Supplementary material for: Canopy Homolog 2 as a Novel Molecular Target in Hepatocarcinogenesis
Source: Cancers (Basel). 2021 Jul 19;13(14):3613. doi: 10.3390/cancers13143613 (PMC8306219; doi:10.3390/cancers13143613)
Supplement: Supplementary file 1 [file cancers-13-03613-s001.zip › cancers-1284641--suppl-update.pdf]

# Supplementary Materials: Canopy Homolog 2 as a Novel Molecular Target in Hepatocarcinogenesis

**Anna Kakehashi, Shugo Suzuki, Masayuki Shiota, Nina Raymo, Min Gi, Taro Tachibana, Vasily Stefanov and Hideki Wanibuchi**

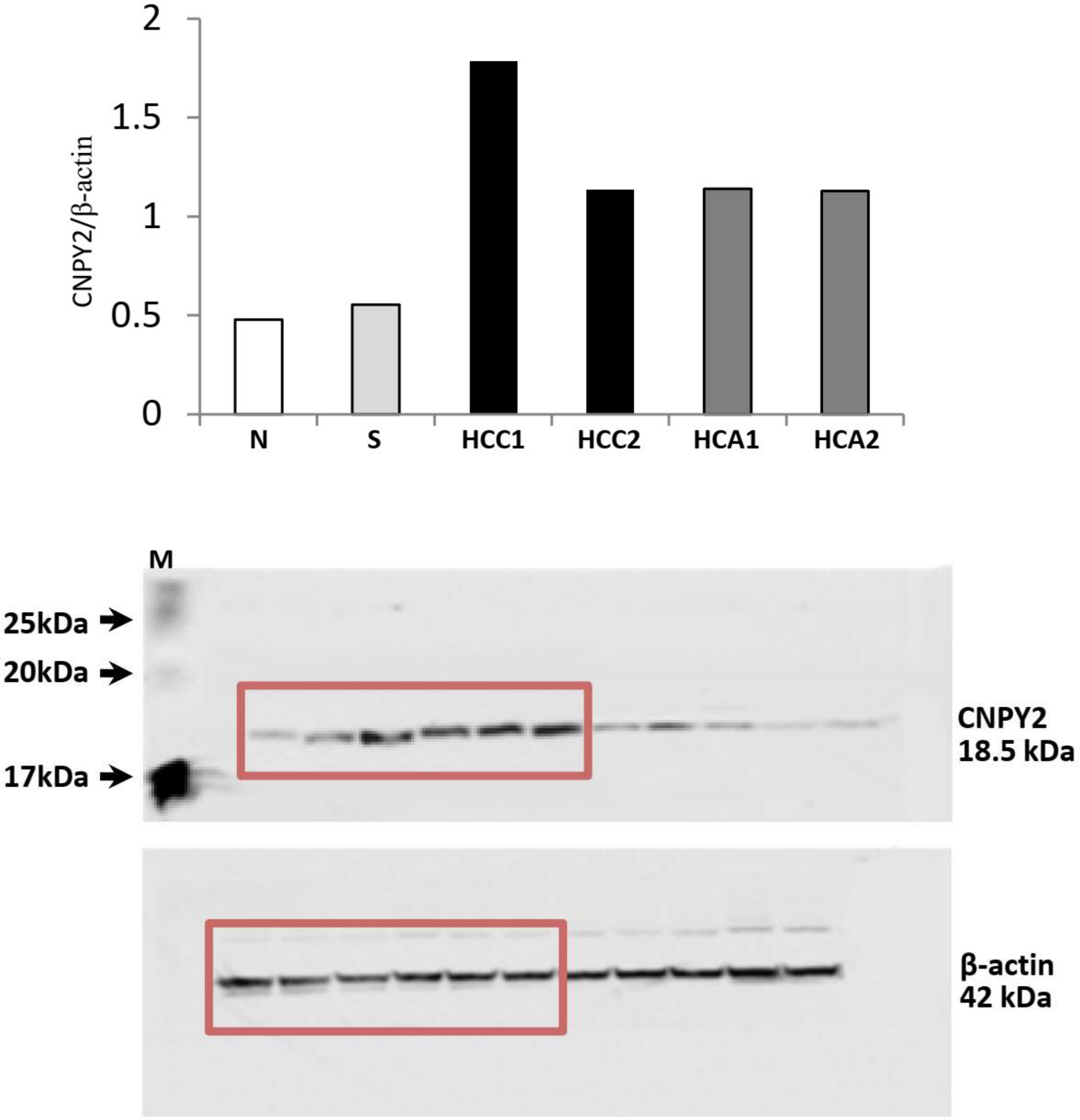


**Figure S1.** Overexpression of CNPY2 in HCCs of DEN-treated C57Bl/6J mice detected by Western blot analysis. Note very low expression of CNPY2 in non-treated mice livers, small increase in non-healthy surrounding livers and high expression in HCCs. N: normal liver; S: surrounding liver.

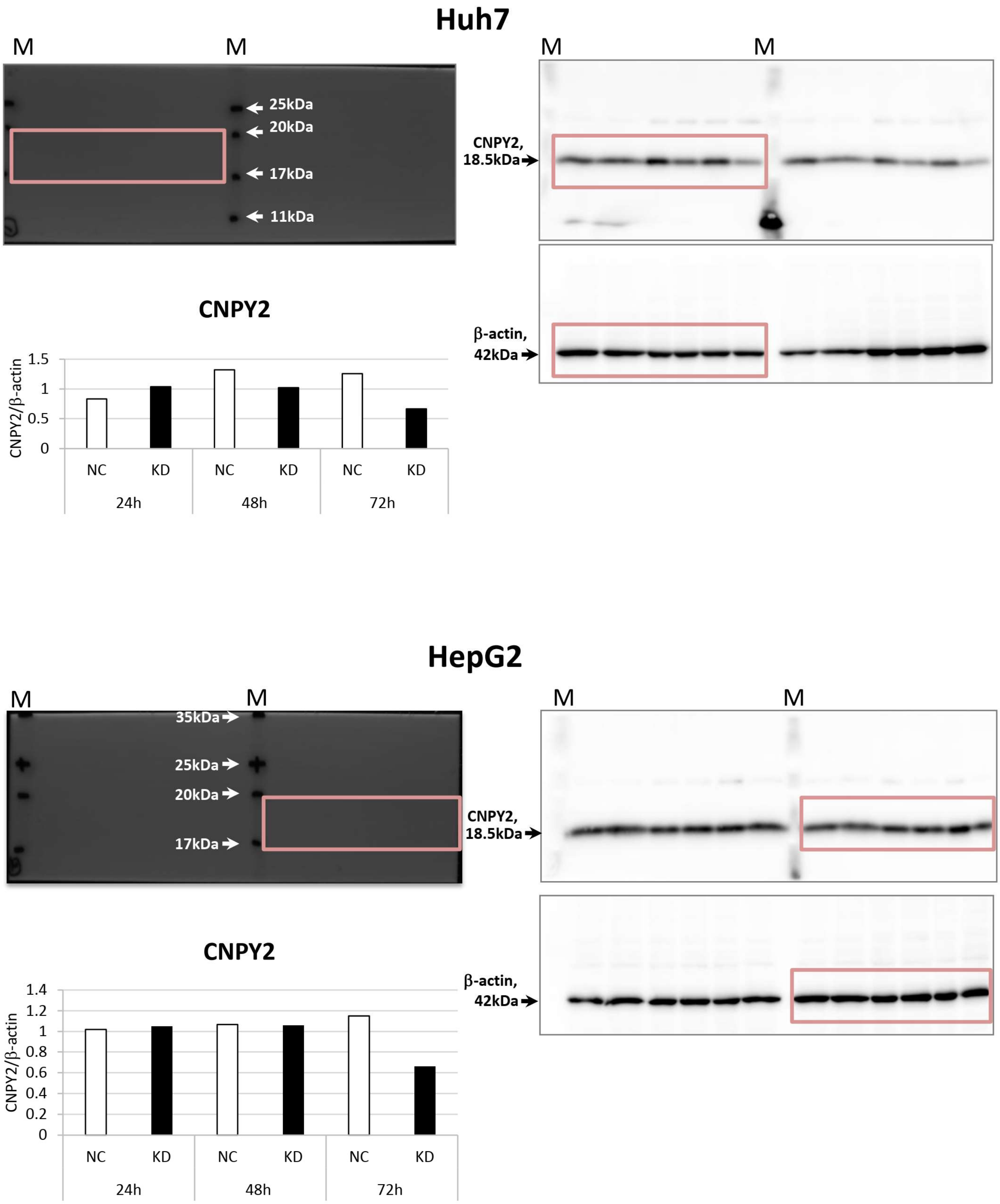


**Figure S2.** CNPY2 knockdown in Huh7 and HepG2 cells detected by Western Blot analysis.

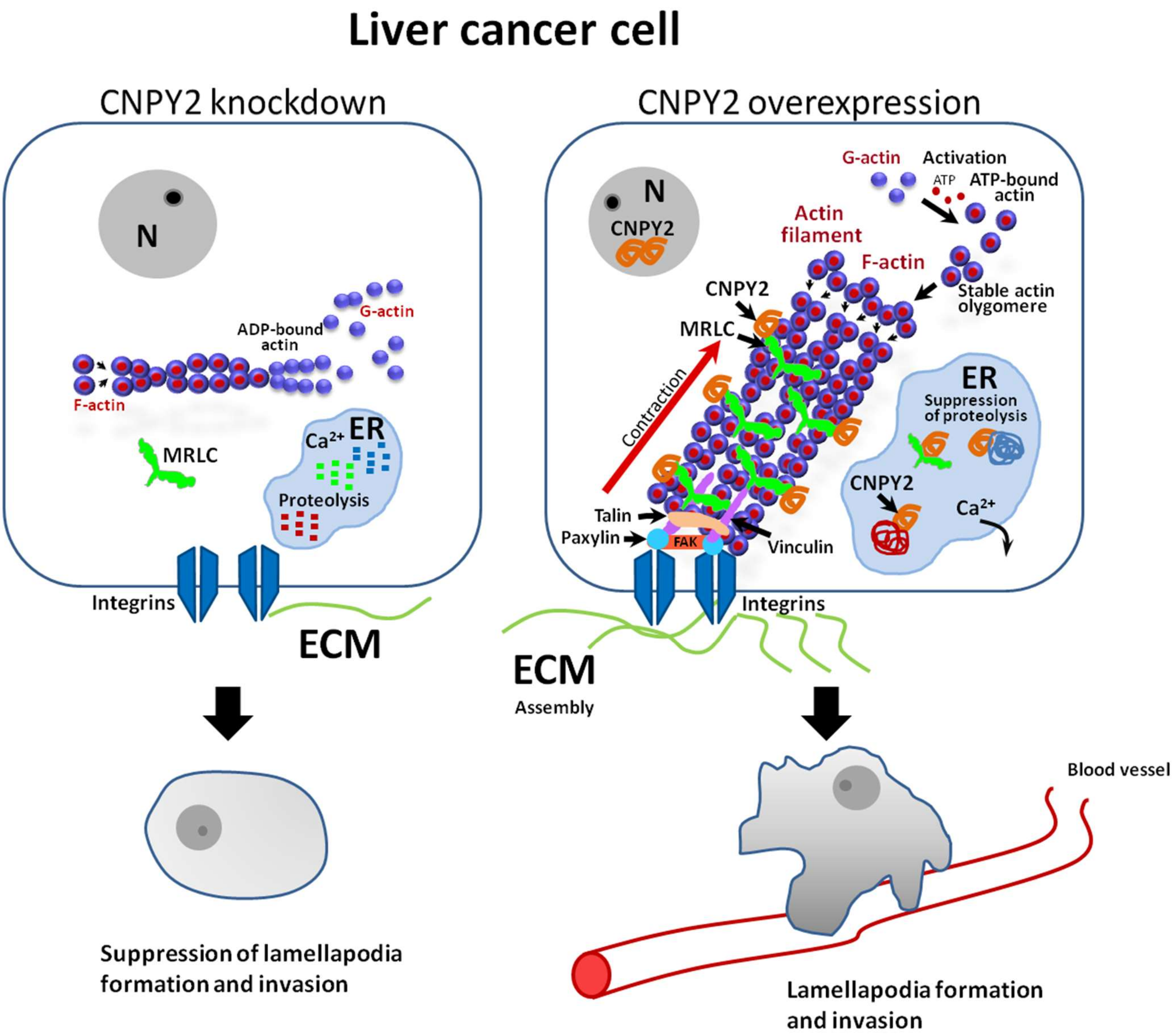


**Figure S3.** A proposed effect of CNPY2 knockdown on ER stress, actomyosin-ECM system and invasion activity of liver cancer cells. N: nuclear; ER: endoplasmic reticulum, ECM: extracellular matrix; G-actin, globular actin; F-actin, filamentous actin; MRLC, myosin regulatory light chain.

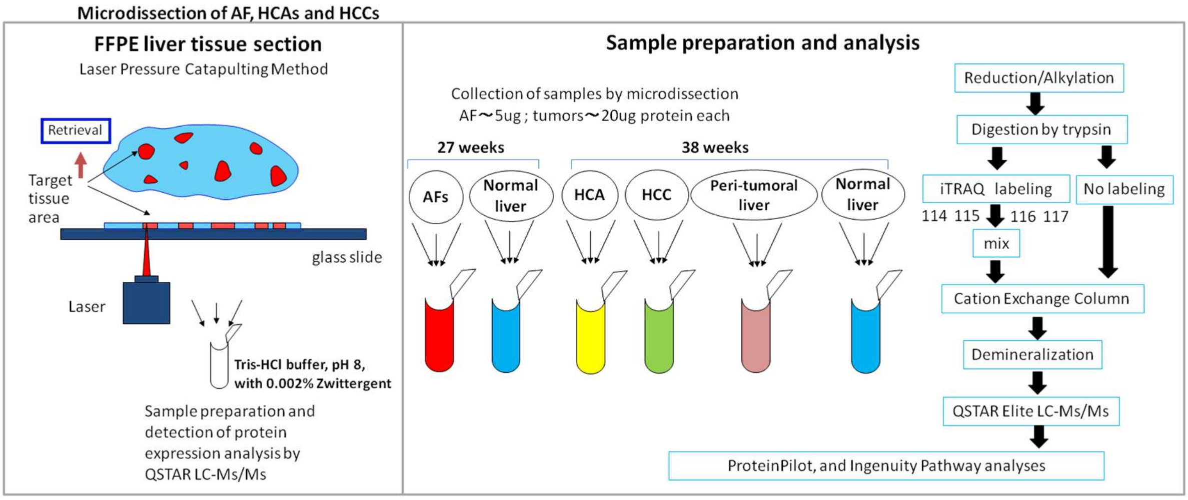


**Figure S4.** Developed method for sensitive proteome analysis in laser-microdissected samples of altered foci and liver tumors of DEN-treated and control C57Bl/6J mice.

**Table S1.** Up-stream regulator analysis in CNPY2kn Huh7 cell line (IPA).

| Upstream Regulator | Molecule Type | Predicted Activation State | Activation z-Score | *p*-Value of Overlap | Target Molecules in Dataset |
|---|---|---|---|---|---|
| TP53 | transcription regulator | Activated | 2.019 | 2E-09 | ATP5MC1,CALU,CLTA,COL3A1,COMT, DDX39A,EPHX1,G6PD,GAPDH,H2AX,HSP90AA1,HSPA5, IDH2,KRT8,LDHA,MDH2,MYH9,PFN1,PKM, PRDX3,RPS12,SOD2, TALDO1,TIMM44,TMED7, TRPV1,TTN,UBC,UGDH |
| SP1 | transcription regulator | Inhibited | −2.392 | 0.01 | ATP5F1B,BACE2,HSPA5,KRT19,PKM,SOD2,UBC,UGDH |
| Ins1 | other | Inhibited | −2.377 | 0.0058 | ALB,G6PD,GAPDH, LDHA,SOD2,TF |
| CEBPA | transcription regulator | Inhibited | −2.19 | 0.0317 | AKR1C1/AKR1C2,ALB,EPHX1,GAPDH, HSPA5, SOD2 |
| NFE2L2 | transcription regulator | Inhibited | −2.112 | 1E-10 | AKR1C1/AKR1C2,ALDOA,COL3A1,EPHX1,G6PD,HSP90AA1, HSPA9,HYOU1,LMNA,PRDX1,PREP,S100P,SOD2, TALDO1,TXN,UBC, UGDH |
| HNF4A | transcription regulator | Inhibited | −2.042 | 0.0003 | AFP,AHSG,AKR1C1/AKR1C2,ALB,CBX3, CLTA, CUX2,EPHX1,G6PD, GAPDH,HSPA5,KH |

| | | | | | |
|---|---|---|---|---|---|
| | | | | | DRBS1, KRT10,KRT8,LDHA, MDH2,PKM,PRDX5, RTF2, SCFD1,SERPINA1, TEX10,TF, TXN |
| MYC | transcription regulator | Inhibited | −2.343 | 1E-10 | AFP,ALB,ALDOA,CANX,COL3A1,G6PD, GAPDH, GOT2,HSP90AA1,HSPA9,IDH2,KIF16B,LDHA, NCL,PKM,PRDX3,PRDX4,PREP,RPL13,RPL28,RPL4,RPLP2, RPS12,SERPINA1,SOD2,TF,TXN |
| HNF1A | transcription regulator | | −1.981 | 0.0142 | AFP,AHSG,AKR1C1/AKR1C2,ALB,COL3A1,HSPA5, SERPINA1 |
| Ige | complex | | −1.98 | 0.0947 | HSPA9,MARCKSL1, PFN1,PRDX1 |
| SREBF1 | transcription regulator | | −1.969 | 0.0021 | ALDOA,G6PD,HSPA5,SERPINA1,TF,TTN |
| PGR | ligand-dependent nuclear receptor | | −1.969 | 0.0037 | COMT,HSPA2,S100P, SERPINA1,SLC9A3R1,RSF7 |
| IGF1 | growth factor | | −1.813 | 0.001 | AKR1C1/AKR1C2,COL3A1,GAPDH,H2AX,HSPA5,LDHA, PKM,TF,TRPV1 |
| MTOR | kinase | | −1.725 | 0.0027 | LDHA,MDH2,PFN1, PKM,PRDX5,SERPINA1,SND1 |
| PCGEM1 | other | | −1.704 | 2E-09 | ALDOA,G6PD,GAPDH,IDH2,LDHA,PKM,TALDO1 |

© 2000-2020 QIAGEN. All rights reserved.

**Table S2.** Patient clinicopathological characteristics, tumor characteristics, and treatment characteristics for HCV+ HCC patients ($N$ = 90).

| Factor | | Median (Range) | $N$ | Percentage |
|---|---|---|---|---|
| **Patient Demographics** | | | | |
| Gender | Male | | 62 | 69 |
| | Female | | 28 | 31 |
| Cirrhosis stage | 1 | | 22 | 24 |
| | 2 | | 19 | 21 |
| | 3 | | 17 | 19 |
| | 4 | | 32 | 36 |
| Age (y) | >70 | 74.04 (71–84) | 22 | 24 |
| | ≤70 | 63.83 (45–70) | 78 | 86 |
| BMI | 15–24 | 21.00 (15.7–24.7) | 75 | 83 |
| | >25 | 26.80 (25.2–28.8) | 15 | 17 |
| Recurrence | Positive | | 54 | 60 |
| | Negative | | 36 | 40 |

| | | | | |
|---|---|---|---|---|
| Stage | I | | 25 | 28 |
| | II | | 41 | 45 |
| | III | | 21 | 24 |
| | IV | | 3 | 3 |
| **Tumor characteristics** | | | | |
| Differentiation | Well | | 8 | 9 |
| | Moderate | | 35 | 39 |
| | Poor | | 47 | 52 |
| Venous invasion | Positive | | 25 | 28 |
| | Negative | | 65 | 72 |
| Depth of tumor invasion | T1 | | 25 | 28 |
| | T2 | | 42 | 47 |
| | T3 | | 20 | 22 |
| | T4 | | 3 | 3 |
| Distant metastasis | Positive | | 1 | 99 |
| | Negative | | 89 | 1 |
| Tumor size ($cm^3$) | ≤2 | 0.7 (0.03–1.95) | 55 | 61 |
| | >2 | 13.5 (2.02–70.31) | 35 | 39 |
| Formation of capsule | Present | | 67 | 74 |
| | Absent | | 23 | 26 |
| Infiltration to capsule | Positive | | 54 | 60 |
| | Negative | | 36 | 40 |
| **Treatment characteristics** | | | | |
| Neoadjuvant therapy | Yes | | 26 | 29 |
| | No | | 64 | 71 |
| Radiation therapy | Yes | | 14 | 16 |
| | No | | 76 | 84 |
| Surgical resection | Lobe | | 9 | 10 |
| | Wedge | | 81 | 90 |

Tumor volume was counted as length/2*(width/2)*(width/2) and expressed in $cm^3$.
